# A Rare Case of Takotsubo Cardiomyopathy Responsive to Fludrocortisone in Secondary Adrenal Insufficiency

**DOI:** 10.7759/cureus.104591

**Published:** 2026-03-03

**Authors:** Waqar Khalid, Muhammad Usman Khan, Maheen Khoso, Nabia Zahid, Naveed Latif, Ali Hassan

**Affiliations:** 1 Anesthesiology and Critical Care, National Hospital and Medical Center, Lahore, PAK; 2 Internal Medicine, East Lancashire Hospitals NHS Trust, Blackburn, GBR; 3 Internal Medicine, National Hospital and Medical Center, Lahore, PAK

**Keywords:** adrenal insufficiency, broken heart syndrome, drug-induced adrenal insufficiency, fludrocortisone, takotsubo cardiomyopathy

## Abstract

We present a case of a middle-aged woman with a prolonged history of diarrhea, vomiting, and abdominal pain. Upon admission to the ICU, she exhibited hypotension and hypoglycemia. Based on presenting gastrointestinal complaints, she was initially treated for acute gastroenteritis; however, continuous refractory hypotension led to an echocardiographic assessment. A left ventricular ejection fraction (LVEF) of 30-35% with apical akinesis and distinct apical ballooning was visualized on a transthoracic echocardiogram, suggestive of takotsubo cardiomyopathy. An increasing inotropic demand raised concerns regarding alternative etiologies. Further evaluation revealed decreased adrenocorticotropic hormone and cortisol levels, leading to a diagnosis of secondary adrenal insufficiency. Treatment with fludrocortisone significantly improved her adrenal insufficiency and her cardiomyopathy. This case highlights secondary adrenal insufficiency as a rare but potential cause of takotsubo cardiomyopathy in Pakistan, with the use of fludrocortisone as a treatment option in a carefully selected patient.

## Introduction

Takotsubo cardiomyopathy, or famously "broken heart syndrome," is a transient left ventricular failure with the absence of ischemic coronary lesions. It typically presents with symptoms like chest pain and dyspnea, just like acute coronary syndrome, preceded by an emotional or physical trigger [1]. Although the exact pathophysiology remains poorly understood, various mechanisms, including catecholamine surge, microvascular dysfunction, and epicardial spasm, have been proposed [2].

While relatively rare, secondary causes of takotsubo cardiomyopathy are crucial to identify, particularly in cases of endocrine disorders such as adrenal insufficiency [3]. In cases of cardiac myopathy secondary to adrenal insufficiency, glucocorticoid and mineralocorticoid replacement therapy have been shown to correct the underlying hormonal imbalance and improve cardiac function [4].

Here, we present a case of takotsubo cardiomyopathy secondary to adrenal insufficiency in a middle-aged female, due to prolonged use of topical steroid cream for a skin rash. This case highlights adrenal dysfunction as one of the triggers for takotsubo cardiomyopathy and suggests fludrocortisone as a potential adjunctive treatment in carefully selected patients.

## Case presentation

A 44-year-old woman with an established diagnosis of carbamazepine-responsive trigeminal neuralgia presented with altered sensorium, refractory hypotension, and recurring hypoglycemia. She had a prolonged history of diarrhea, nausea, and abdominal pain with a history of use of topical steroid cream for a chronic rash. The patient had been frequently using clobetasol propionate (0.05%) for four months before arrival in the emergency department. On physical examination, she had a heart rate of 144 beats per minute (bpm), blood pressure of 97/53 mmHg, and a shock index of 1.5. She was lethargic and drowsy but arousable with a GCS of 14/15. The blood tests on admission showed hyponatremia, hypokalemia, and an increased CRP (Table 1). The hypokalemia was initially attributed to gastrointestinal potassium loss via diarrhea, with a possibility of compounded effect by prolonged steroid-induced potassium wasting.

**Table 1:** Blood result tests on admission. WBC: white blood cells; CRP: C-reactive protein; ALT: alanine aminotransferase; PLT: platelets; AST: aspartate aminotransferase

Lab tests	Values	Reference range
Hemoglobin (g/dL)	12	11.5-15.0
WBC count (k/μL)	10.2	4-11
PLT (k/μL)	183	150-400
CRP (mg/dL)	4.73	<0.5
Sodium (mmol/L)	130	136-145
Potassium (mmol/L)	2.8	3.5-5.1
Chloride (mmol/L)	108	98-107
ALT (U/L)	15	<34
AST (U/L)	19	<31
Urea (mg/dL)	36	10-50
Creatinine (mg/dL)	0.59	0.55-1.02

**Figure 1:**
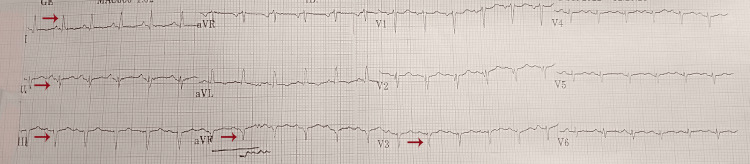
Electrocardiogram showing NSR at 144 bpm, LAD, LAFB, poor R-wave progression in the precordial leads, and no acute ST-T segment changes. LAD: left axis deviation; NSR: normal sinus rhythm; BPM: beats per minute; LAFB: left anterior fascicular block

The patient's preliminary ECG showed a normal sinus rhythm with a heart rate of 144 beats per minute (bpm), and poor R-wave progression in precordial leads (Figure 1). Initially, the patient was managed for gastroenteritis-induced hypovolemia secondary to sepsis (qSOFA 3) with crystalloids, but she remained hypotensive, and this necessitated an escalating requirement of multiple vasopressors along with supplemental hydrocortisone. The patient also started experiencing runs of non-sustained ventricular tachyarrhythmias, which prompted a cardiology consultation. A thorough cardiac workup revealed slightly elevated troponins and distinct apical ballooning, with akinesis of the mid-to-apical anteroseptal and lateral left ventricle segments (Table 2). This was associated with a moderately reduced global systolic dysfunction (left ventricular ejection fraction {LVEF}: 30-35%), suggestive of takotsubo cardiomyopathy on echocardiogram. At this point, coronary angiography was advised; however, the patient's family declined the procedure. Consequently, further management was done with a working diagnosis of ‘probable takotsubo’ cardiomyopathy.

Vasopressor and steroid-resistant hypotension, with recurrent hypoglycemia and a history of chronic exogenous topical steroids, led to a high suspicion of adrenal insufficiency. Borderline low serum cortisol despite being treated with hydrocortisone and low adrenocorticotropic hormone (ACTH) levels confirmed our diagnosis of adrenal insufficiency (Table 2). Takotsubo cardiomyopathy was attributed to secondary adrenal insufficiency, likely due to chronic excessive topical steroid use.

**Table 2:** Laboratory tests conducted in intensive care unit. ACTH: adrenocorticotropic hormone

Lab tests	Values	Reference range
Plasma troponin I (ng/mL)	0.533	0-0.015
Plasma ACTH (pg/mL)	<1.00	7.3-63.3
Cortisol (µg/dL)	3.9	3.70-19.40

She was started on fludrocortisone to manage both her adrenal insufficiency and persistent hypotension, with cautious monitoring of her electrolytes, vascular volume status, and follow-up echocardiograms. On the 16th day of admission, the patient showed a complete de-escalation of vasopressors and marked improvement in left ventricular ejection fraction (LVEF: 45-50%), with only mildly hypokinetic mid-to-apical anteroseptal left ventricle segments. This improvement indicated that fludrocortisone had a positive influence on the underlying triggers and cardiac function. She was later discharged on oral fludrocortisone and referred to endocrinologists for further evaluation.

## Discussion

This case emphasizes secondary adrenal insufficiency as one of the causative factors for takotsubo cardiomyopathy, particularly in patients with atypical features such as steroid-refractory hypotension and hypoglycemia. Adrenal insufficiency, whether primary or secondary, can lead to cortisol and, in some cases, aldosterone deficiency, resulting in hemodynamic instability and metabolic disturbances that can affect the cardiovascular system [5]. In a patient with adrenal insufficiency, cortisol deficiency may impair catecholamine signaling, leading to reduced β-adrenergic receptor expression, diminished vascular smooth muscle responsiveness, and decreased myocardial calcium-handling efficiency. Catecholamine resistance, despite relatively elevated endogenous catecholamines in takotsubo cardiomyopathy, results in reduced systemic vascular resistance, impaired inotropy, and a tendency toward refractory hypotension. The catecholamine surge observed in takotsubo cardiomyopathy may exacerbate these effects, leading to myocardial stunning and dysfunction [6].

The diagnosis of takotsubo cardiomyopathy due to secondary adrenal insufficiency in our patient was supported by the characteristic echocardiographic findings of apical akinesia and ballooning, consistent with previous reports [3,7]. Positive response to treatment with fludrocortisone under electrolyte monitoring further backed the consideration of adrenal dysfunction in the pathogenesis of takotsubo cardiomyopathy [8]. While we emphasize the role of fludrocortisone in the clinical improvement of our patient with takotsubo cardiomyopathy, it is important to remember that fludrocortisone can itself precipitate takotsubo cardiomyopathy if given at supraoptimal doses [9].

Only a handful of cases of takotsubo cardiomyopathy associated with secondary adrenal insufficiency in adults have been reported [3,10]. It has been suggested that cortisol deficiency may impair myocardial contractility and vascular responsiveness, while aldosterone deficiency may contribute to fluid and electrolyte imbalances, both of which can predispose to takotsubo cardiomyopathy [11].

In addition to conventional diagnostic methods, emerging work, such as the LAD Electromechanical Mismatch Index, highlights that electromechanical signatures and multimodal assessments may be leveraged for diagnosis and prognostication, especially in cases with rare clinical presentations [12]. Socioeconomic status, limited access to healthcare, and chronic psychosocial stress play a significant role in shaping cardiovascular risk. These factors influence not only the prevalence of traditional cardiovascular risk factors but also stress physiology and inflammatory burden, all of which are relevant to stress-mediated cardiac conditions such as takotsubo cardiomyopathy [13]. By coupling these novel diagnostic indices with an appreciation of the socioeconomic determinants of health, we can strengthen our understanding of why certain populations are more vulnerable to stress-mediated myocardial injury.

## Conclusions

Our case explores the association between secondary adrenal insufficiency and takotsubo cardiomyopathy, emphasizing the need for greater awareness of rare causes of this syndrome. When patients present with symptoms indicative of takotsubo cardiomyopathy, clinicians should explore adrenal insufficiency as a causative factor where relevant, especially in the presence of other atypical symptoms such as gastrointestinal disturbances and hypoglycemia.

This case demonstrates that, in selected clinical scenarios, particularly when effective circulatory volume is compromised and cardiac reserve is limited, mineralocorticoid therapy with fludrocortisone may offer a physiologically advantageous adjunct therapy. Further research and clinical focus are essential for a better understanding of the relationship between secondary adrenal insufficiency and heart failure in order to improve early diagnosis and management in affected patients.
